# On the Piezoelectric Detection of Guided Ultrasonic Waves

**DOI:** 10.3390/ma10111325

**Published:** 2017-11-18

**Authors:** Kanji Ono

**Affiliations:** Department of Materials Science and Engineering, University of California, Los Angeles (UCLA), Los Angeles, CA 90095, USA; ono@ucla.edu; Tel.: +1-310-825-5534

**Keywords:** guided ultrasonic waves, detector calibration, receiving sensitivity, Lamb waves, acoustic emission

## Abstract

In order to quantify the wave motion of guided ultrasonic waves, the characteristics of piezoelectric detectors, or ultrasonic transducers and acoustic emission sensors, have been evaluated systematically. Such guided waves are widely used in structural health monitoring and nondestructive evaluation, but methods of calibrating piezoelectric detectors have been inadequate. This study relied on laser interferometry for the base displacement measurement of bar waves, from which eight different guided wave test set-ups are developed with known wave motion using piezoelectric transmitters. Both plates and bars of 12.7 and 6.4 mm thickness were used as wave propagation media. The upper frequency limit was 2 MHz. Output of guided wave detectors were obtained on the test set-ups and their receiving sensitivities were characterized and averaged. While each sensitivity spectrum was noisy for a detector, the averaged spectrum showed a good convergence to a unique receiving sensitivity. Twelve detectors were evaluated and their sensitivity spectra determined in absolute units. Generally, these showed rapidly dropping sensitivity with increasing frequency due to waveform cancellation on their sensing areas. This effect contributed to vastly different sensitivities to guided wave and to normally incident wave for each one of the 12 detectors tested. Various other effects are discussed and recommendations on methods of implementing the approach developed are provided.

## 1. Introduction

In structural health monitoring (SHM) and nondestructive evaluation (NDE) fields, guided ultrasonic waves are widely utilized [1,2]. In ultrasonic, acoustic emission (AE) and acousto-ultrasonic testing of large tank or piping structures, Lamb (or plate) waves are the dominant wave propagation modes [3,4]. Lamb waves have been studied for more than a century [5,6] and utilized for industrial applications [7,8]. While the surface displacement (or velocity) of such waves can be characterized without contact using laser instruments in well-equipped laboratories, practical detection methods ordinarily use piezoelectric detectors, especially for low pm-level signals. For a typical laser interferometer, displacement needs to be above 0.1 nm (and less than 20 nm). Besides, it is still expensive and requires a stable optical platform. Basic piezoelectric transducers (for transmitting and receiving) and sensors (for receiving) are constructed from a piezoelectric disc with a protective front face and a backing material for suppressing unwanted vibration. Most manufacturers have supplied industrial ultrasonic transducers and AE sensors with a certificate with a pulse-echo response or with a relative performance chart to a reference standard. These are for normally incident waves (NIW) of longitudinal mode. For this type of waves, conventional face-to-face calibration methods have recently been demonstrated to provide receiving sensitivity in absolute scale. This new approach utilized a laser interferometer as the basis of displacement calibration [9]. Here, pulse-excited transmitters were characterized for their displacement outputs, which were then used to drive receiving sensors in contact, culminating in their receiving sensitivities by spectral subtraction. A similar approach was next developed to evaluate sensor responses to guided waves, although it was limited to bar waves [10]. It was shown that displacement sensitivities to NIW and to guided waves were always different for 16 sensor types tested. Bar wave sensitivities always rose at low frequencies. This study pointed to the need for an expanded work since guided waves vary when the geometries of a wave propagating medium change. The geometrical effects can also be used in designing special waveguides to compensate for the dispersion phenomena, as demonstrated by de Marchi et al. [11]. Thus, it is necessary to examine the behavior of sensor responses to guided waves under multiple conditions. In the present study, both plates and bars are used in two thicknesses each. Another factor introduced is the method of wave excitation in symmetric or asymmetric manner. That is, eight different conditions are utilized. Displacement sensitivities for 12 different sensors were obtained. While variations were large in the sensitivity spectra, reflecting selective wave propagation modes, unique averaged behavior emerged in all cases evaluated implying the presence of underlying sensor characteristics. Results will be discussed from basic and practical aspects.

Uses of plates and bars in AE sensor validation have a long history. A plate material was introduced to the CARP procedures for fiber-reinforced plastics using AE [12]. Here, CARP stands for the Committee on Acoustic Emission from Reinforced Plastics, the Society of the Plastics Industry (New York, NY, USA). A lead sheet of 1.22 m × 1.83 m × 13 mm was initially specified for wave propagation medium to set the threshold level of AE detection. A mild steel bar (3 m × 51 mm × 19 mm) was also used for validating reference amplitude threshold of strong AE signals. A slightly smaller lead plate was used in standards published in 1985 [13,14]. Pencil-lead breaks were the source of AE signals. Later, the lead plate material was replaced by an acrylic rod of 788 mm length and 38 mm diameter [15,16]. These uses of plates and bars, however, have been for relative and indirect measurements of sensor sensitivity.

There are several related works of interest on Lamb waves and bar waves. Dunegan [17] conducted wave propagation experiments using 12.5 and 6.3 mm-thick bars and developed a strategy of separating crack-induced AE signals from noise. Waveform analysis was the primary tool in his work. Hamstad and coworkers made a series of 3-dimensional dynamic finite-element calculations, examining Lamb wave propagation [18,19,20,21]. Using a simulated pencil-lead break source, they calculated resultant waveforms and their frequency spectra for various source and propagation conditions. Since the use of pencil-lead breaks introduces repeatability question in practice, it requires a reference measurement in applications for sensor calibration. This last part was difficult to implement on plates and bars, preventing further development. These Lamb wave studies were intended for improving AE source location accuracies. In this connection, a recent study of introducing probabilistic analysis in combination with Kalman filtering is of interest [21] as well as a new principle of Lamb wave detection with nonlinear phononic crystals [22].

Before embarking on Lamb wave sensing, it is necessary to distinguish two types of waves detected by AE sensors; normally incident waves or NIW and guided waves. For the latter, wave motion normal to the surface is usually detected while the guided waves travel along the surface. Further note that sensor sensitivities to NIW and bar waves differed completely when compared in [10]. Even when NIW sensitivity was flat, bar-wave sensitivity had sharply rising behavior at lower frequencies. Most commonly dealt type in sensor calibration is for NIW on the sensor face since this type of waves are produced in thick-walled structures, arriving at surface-mounted sensors. Sensor calibration for this type can best be conducted by face-to-face arrangement, as reported in [9], but no national or international standard exists. This topic was further investigated in a recent study [23], which demonstrated that reciprocity sensor calibration methods as presently used in Japan [24] have no valid foundation. For guided wave sensors, a surface-wave calibration method was developed at National Institute of Standards and Technology (NIST) and became an international standard [25]. At present, however, NIST offers no calibration service. Consequently, no primary calibration for AE sensor is available anywhere globally. Lamb waves and bar waves are utilized widely in SHM and NDE fields as noted at the start, yet no calibration methods have been studied until recently for bar waves [10,26]. These works were still limited in the propagation medium used and more comprehensive approach is needed to eventually develop them into practical AE sensor calibration procedures. These are essential elements of SHM and NDE technology, but are still unavailable today.

The approach here is to use repeatable wave sources coupled to laser interferometry in providing the basis for sensor calibration for the detection of guided waves, both Lamb and bar waves. This goal is accomplished and this work has successfully quantified the wave motion of guided waves for plates and bars and determined the guided-wave receiving sensitivities of AE sensors and ultrasonic transducers in absolute physical units. A scheme for practical calibration procedures is also described.

## 2. Experimental Procedures

In order to determine the receiving sensitivity of an ultrasonic transducer or an AE sensor to guided waves, such waves need to be generated reproducibly. Two aluminum plates, 12.7 mm × 1220 mm × 1220 mm and 6.4 mm × 308 mm × 1000 mm, and three aluminum bars, 6.4 mm × 25.4 mm × 3600 mm, 6.4 mm × 25.4 mm × 1070 mm and 12.7 mm × 50.8 mm × 1220 mm were used as wave propagation media. The material was UNS A96061 (6061-T6511 temper from Kaiser Aluminum, Spokane, WA, USA) with the mass density of 2.70 Mg/m^3^, the longitudinal wave velocity = 6.42 mm/µs, and the transverse wave velocity = 3.04 mm/µs. The longest aluminum bar was bent to allow transport for laser interferometry and was previously described [10]. A brief description of bar wave set-ups is given in Appendix. One edge of each piece was polished flat. For the plates, the polished parts were about 150 mm wide in the middle. The positions of sensor placement were also polished on the surface of the plates or bars. An ultrasonic transducer was affixed by epoxy glue at a central position of the polished edge. Four FC500 transducers (Acoustic Emission Technology Corp, Sacramento, CA, USA) and a V104 transducer (Olympus, MA, USA) were used as transmitters. These were previously characterized [9,10]. For sensor testing, one of the mounted transducers was driven by a pulse generator as reported previously [10]. For the bent bar, the normal displacement due to bar waves was measured using a laser interferometer (Thales Laser, LH-140, Orsay, France) at distance of 300 mm from the bar end and the results were given previously [10]. For this bent bar set-up, the same FC500 transducer has been kept in the glued state since the laser measurement.

**Figure 1:**
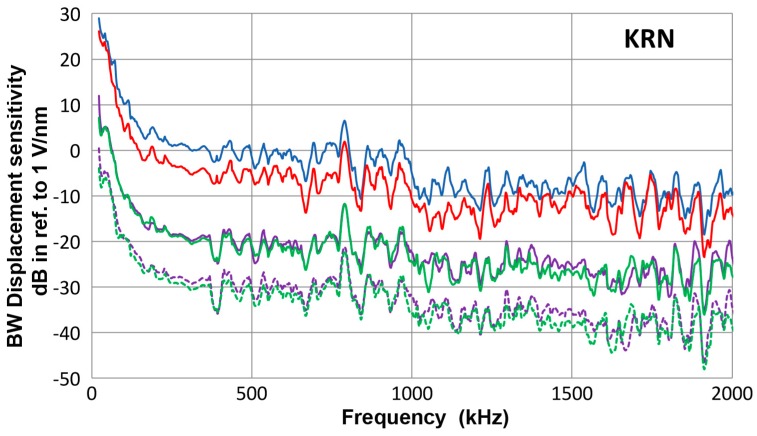
Bar-wave receiving sensitivities of KRN sensors. **Blue**-#11053, **red**-#11063, **green**-#12060, **purple**-#12035 (solid curves with 10 MΩ and dashed curves with 100 kΩ).

Normal displacements due to guided waves in the plates and shorter bars were characterized using four point-contact sensors (two each of types KRNBB-PC and –PCP, KRN Services, Richland, WA, USA). Their bar-wave receiving sensitivities in terms of the magnitude fast Fourier transform (FFT) spectra in reference to 0 dB at 1 V/nm were obtained in [10] and are reproduced in Figure 1. As noted before, the sensitivity increased by about 30 dB below 300 kHz with decreasing frequency. Here, the data for KRN sensors without field effect transistor (FET) buffer (type KRNBB-PCP) were taken with higher input resistance of 100 kΩ or 10 MΩ (see Appendix D of [10] for details). The previous input resistance of 10 kΩ produced an unwanted high-pass filtering effect. KRN sensors of type KRNBB-PC were used with a supplied power supply (KRN AMP 1BB-J). By placing one of the KRN sensors at 300 mm (150 mm for the 6.4 mm-thick plate) from the driving transducer surface, output voltages corresponding to the normal displacements due to guided waves are obtained. These signals were digitized at 2 ns time interval and recorded using a Pico Scope (Pico Tech 3405A, Pico Technology, St. Neots, UK).

Two excitation methods were used. One is for symmetric excitation, where a transmitter was positioned centrally on the plate or bar end. This was previously used in [10], producing mostly symmetric guided-wave modes. Some slow-moving asymmetric modes were detected at wave velocities below 1.8 mm/µs when KRN sensors were used. These modes were undetected by laser interferometry, however. The other method for activating asymmetric modes was introduced in Appendix A of [10]. This method placed a square-rod of 3.2 mm on the lower edge face. Most wave modes generated were asymmetric guided wave modes. For the case of 12.7 mm-thick plate, the square rod was also placed at the edge on the top surface since the side excitation produced weaker wave intensities. Final results were identical, but the top excitation, also known as an out-of-plane source [16,17], provided a higher signal-to-noise ratio.

When the displacement data is obtained, a transducer or a sensor under test is coupled at the measurement position on the surface of the test plate or bar. Vaseline was used as a couplant as before [9,10]. The output voltages were digitized using the Pico Scope input terminated with 10 kΩ. This value was selected since most AE preamplifiers have the input impedance of 10 kΩ. Data analysis procedures followed those described elsewhere [9,10]. It should be noted that FFT spectra exclude waveform data of late arriving parts at less than 1.5 mm/µs wave velocity.

## 3. Results and Discussion

### 3.1. Normal Displacement

**Figure 2:**
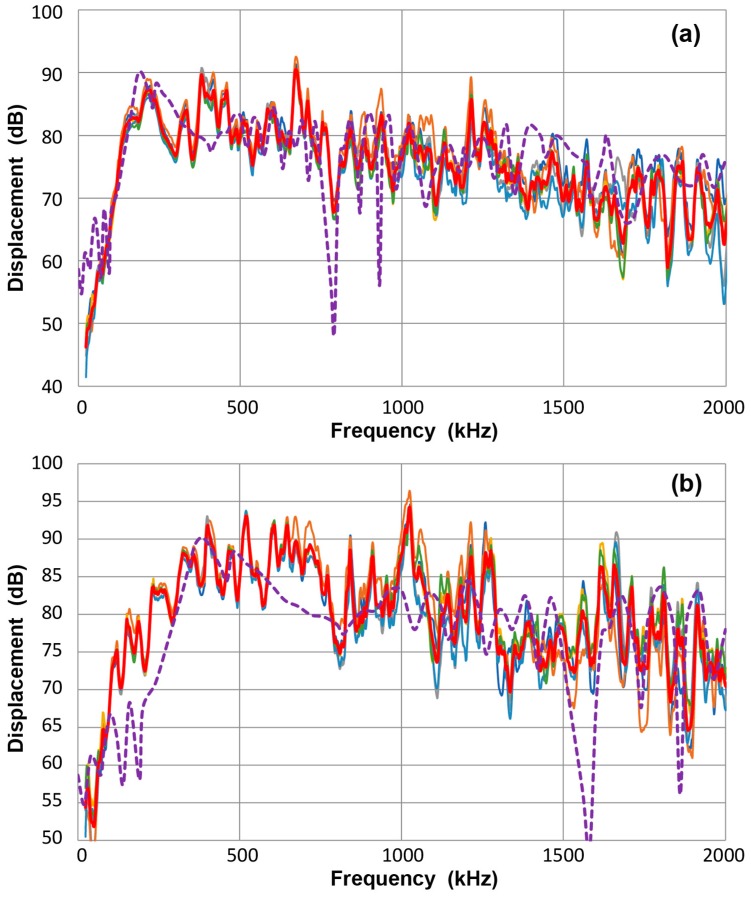
(**a**) FFT magnitude spectrum of normal displacement at 300 mm for 12.7 mm-thick plate with symmetric excitation. Average data is given in **red**. Six others are plotted, but mostly hidden. KRN sensors with FET-**blue** and **orange**, no FET with 100 kΩ-**grey** and **yellow**, with 10 MΩ-**light blue** and **green**. Calculated spectrum from [19] is plotted in **purple** dash curve. (**b**) FFT magnitude spectrum of normal displacement at 300 mm for 6.4 mm-thick bar with asymmetric excitation. Average data is given in red. Six other curves have same color code as in (**a**). Calculated spectrum from [19] is plotted in **purple** dash curve.

Except for the bent bar set-up, for which laser interferometric measurements were available, it is necessary to obtain normal displacement data for seven other plate and bar test set-ups through a secondary calibration relying on the bar-wave displacement sensitivity of KRN sensors. For each set-up, six measurements were taken using KRN sensors. For the non-FET-buffered sensors, two tests each were made varying the input termination resistance between 100 kΩ and 10 MΩ. Figure 2a shows the results for 12.7 mm-thick plate, including the averaged data (in red curve). Six curves mostly overlap each other and this matching behavior to the averaged curve is reflected in a low averaged value of the standard deviation over 22 kHz–2 MHz of 1.8 dB. Here, 0 dB is in reference to 1 nm (except in some FFT representation, this is shifted down by 108.37 dB for the correction of input file length of 256 k-points) [23]. This displacement data indicates a decreasing trend at frequency below 140 kHz and shows low intensity of S_0_-mode Lamb waves at low frequencies. This behavior was predicted by Ichikawa’s calculation [27,28], based on Viktorov theory [5].

Another example is given in Figure 2b. This is for a 6.4 mm-thick bar with asymmetric excitation. The averaged spectrum is shown in red curve. Again, six curves mostly overlap each other and this matching behavior is reflected in a low averaged value of the standard deviation over 22 kHz–2 MHz of 1.8 dB. More fluctuations of displacement are found and a decreasing trend at low frequencies started at 300 kHz. The spectral levels are comparable to Figure 2a as well as to that of the laser interferometer data for the bent bar (also 6.4 mm thick) with symmetric excitation [10].

The observed trend of displacement spectra has features in common with out-of-plane displacements calculated by Hamstad et al. [19]. Their calculations were for steel plates of two thicknesses and used a mid-plane vertical dipole source with a rise time of 1 µs. Travel distance for 25.4 mm-thick plate case was 381 mm. By converting the frequency for thicknesses used in our cases, the calculated spectra are shown in purple dash curves in both figures. The low frequency peak appeared at 190 kHz for the 12.7 mm-thick plate, matching the first peak observed in Figure 2a. The initial slope was also similar between the calculated and observed curves. Surprisingly, many peaks and dips at higher frequencies also coincided, implying that the propagation modes strongly affect the displacements after travel. Hamstad calculation [19] also found 15 dB decrease to 2 MHz, again comparable to observed decrease in Figure 2a. The Hamstad calculation was equivalent to symmetric excitation, but it showed similar spectral features as that observed in the asymmetrically excited 6.4 mm-thick bar, shown in Figure 2b. The calculated and observed displacement spectra follow the same trend except at lower frequencies. While the parameters used for the computational and experimental approaches were different, the similarity of results found here gives confidence in the method used in the present study.

Note that the reported dB-amplitude of the Hamstad calculation ranged from −220 to −188 dB in reference to 1 m displacement. This was converted to 1 nm reference by adding 180 dB. Assuming their FFT used 16,384 points, 84.3 dB was added, making the peak at 76.3 dB. Since the peak level of calculated displacement was around 0.1 nm, this peak FFT amplitude is expected. Additional 14 dB adjustment was made to match the peak values in Figure 2a. This last addition was for an easier comparison of the two spectra.

**Figure 3:**
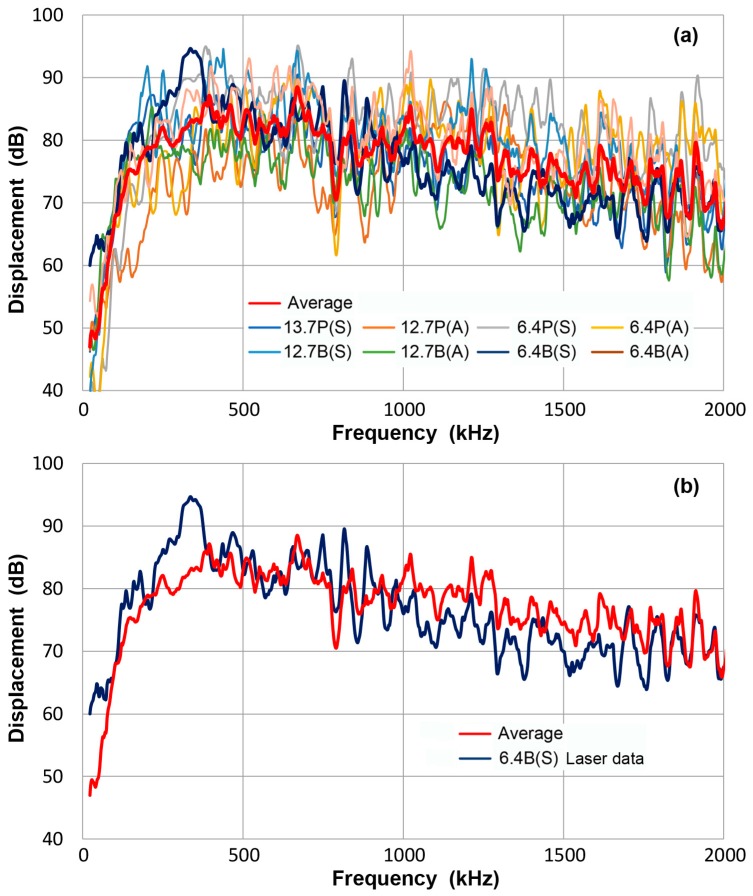
(**a**) FFT magnitude spectrum of normal displacement at 300 mm. Average of eight curves-red, thin bar 6.4B(S)/laser data-dark blue, thick plate 12.7P(S)-blue, thick plate 12.7P(A)-orange, thin plate 6.4P(S)-grey, thin plate 6.4P(A)-yellow, thick bar 12.7B(S)- light blue, thick bar 12.7B(A)-green, thin bar 6.4B(A)-brown. Tests designated by thickness in mm, followed by P or B for plate or bar and (S) or (A) for symmetric or asymmetric excitation. (**b**) Two of the same spectra from (**a**): Average of eight curves-red, 6.4B(S)/laser data-dark blue.

Figure 3a compares all eight displacement spectra, including one laser-based in dark blue curve. The red curve represents the averaged spectrum of the eight spectra. While details cannot be followed, Figure 3a exhibits the overall resemblance among the displacement spectra and the data spreads are within approximately ± 10 dB of the averaged curve. For both symmetric and asymmetric excitation methods, a trend exists for a rapid rise at low frequencies, reaching a peak at mid-frequency range, followed by a gradual decrease to 2 MHz. This finding also implies that the displacement peaks are approximately 0.5 to 1 nm. Figure 3b shows the averaged spectrum (red curve) and laser-based spectrum of the bent bar (dark blue curve). Spectral differences between these two are less than 10 dB.

Ideally, displacement spectra determined here should be verified with laser interferometer measurements. Lacking access to such a facility, these can provide a secondary reference to obtain displacement sensitivities of various transducers and sensors. Note that each displacement spectrum is distinct and needs to be paired with measurements made with an identical condition. The average spectrum is given only to show the general trend of displacement spectra.

**Figure 4:**
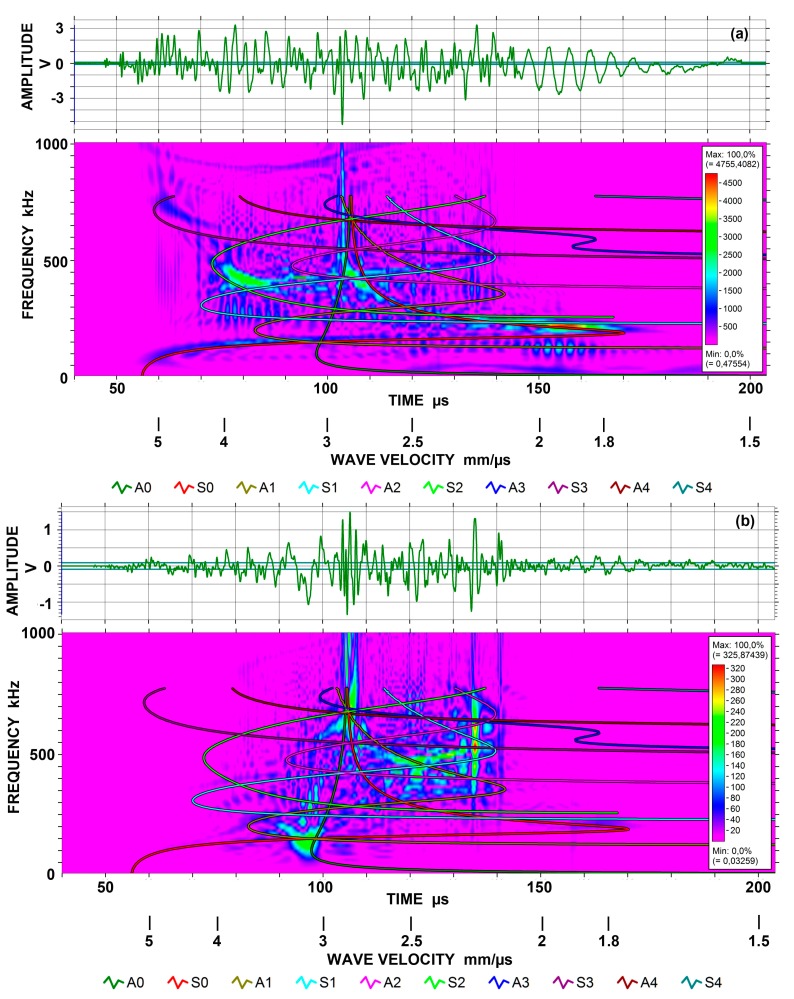
(**a**) Choi-Williams transform spectrogram for symmetrically excited Lamb waves on 12.7 mm-thick plate using KRN sensor. The output of 40–200 µs segment up to 1 MHz shown. (**b**) As (**a**), but for asymmetrically excited Lamb waves on 12.7 mm-thick plate.

**Figure 5:**
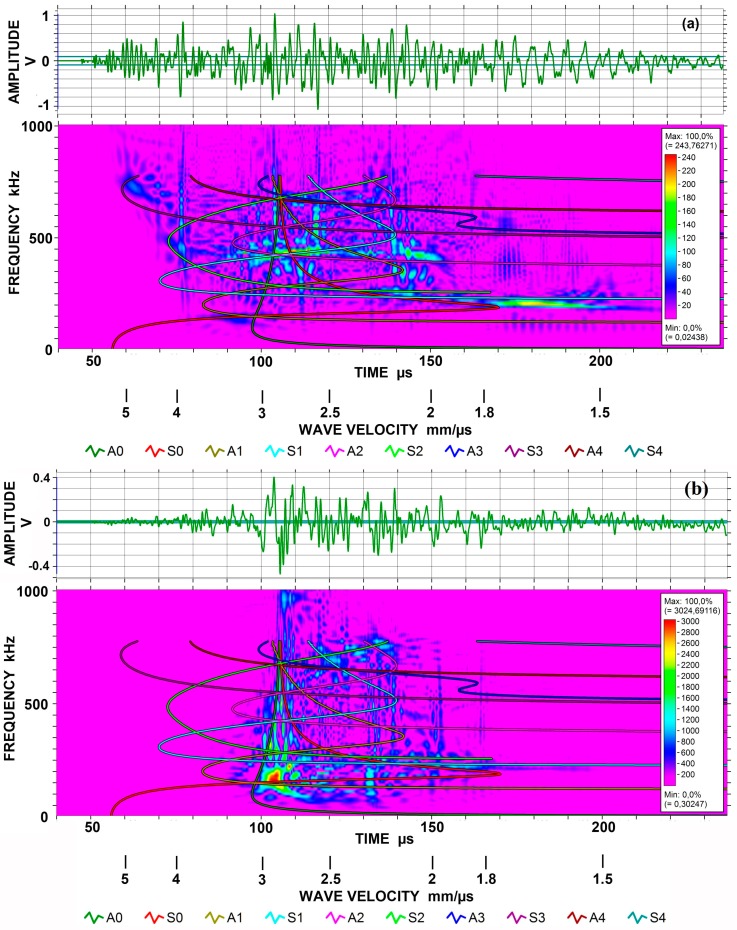
(**a**) Choi-Williams transform spectrogram for symmetrically excited bar waves on 12.7 mm-thick bar using KRN sensor. The output of 40–220 µs segment up to 1 MHz shown. (**b**) As (**a**), but for asymmetrically excited bar waves on 12.7 mm-thick bar.

Wave modes can be seen in spectrograms obtained by Choi-Williams transform (CWT). This CWT used AGU-Vallen Wavelet software (Vallen Systeme, Icking, Germany; version R2017.0504.1). Four examples are given in Figure 4a,b for the 12.7 mm-thick plate and in Figure 5a,b for the 12.7 mm-thick bar, each with symmetric and asymmetric excitation methods. A KRNBB-PC sensor was used for these tests.

Figure 4a is a CWT spectrogram for symmetrically excited Lamb waves. Waveform is shown at the top, stretching from about 50 to 190 µs. Slower arrival beyond 200 µs was of low frequency of ~10 kHz and was ignored. Because the thickness is 12.7 mm, five symmetric modes are present in the frequency range to 1 MHz. Lamb wave dispersion curves for ten modes are overlaid to 800 kHz. One can readily recognize the lowest symmetric Lamb mode of S_0_, starting from 5.5 mm/µs at 50 kHz. The strongest peak is found at 0.2 MHz and 1.8 to 2.1 mm/µs, where S_0_ and S_1_ modes overlap and S_0_ reaches a velocity minimum or velocity trough. As the S_0_ trace moves to the left (up in velocity), another peak occurs at 0.4 MHz and 2.9 mm/µs, where S_0_ and S_1_ modes intersect again. At 0.4 MHz, another peak is found at 4 mm/µs corresponding to the intersection of S_1_ and S_2_ modes. Two faint peaks can be seen at 0.7 MHz, 5 mm/µs and at 1 MHz, 5.4 mm/µs, corresponding to the positions of velocity maxima of S_3_ and S_4_ modes. The largest amplitude in the waveform was at 103 µs, corresponding to the vertical line in the spectrogram. With the velocity of 2.92 mm/µs, this corresponds to Rayleigh wave propagation, as it is slightly faster than the asymptotes of S_o_ and A_0_ modes. Figure 4b shows the waveform and CWT spectrogram for asymmetrically excited Lamb waves. The plate thickness is again 12.7 mm. This plot gives less clear correspondence to the dispersion curves as mode traces are not revealed. However, most high intensity zones appear to be those of asymmetric Lamb modes. The strongest peak stretches from 0.33 to 0.7 MHz at 2.2 mm/µs, corresponding to two velocity troughs of A_1_ and A_2_ modes, passing through another trough of S_1_ mode. In fact, the strongest point occurs at the S_1_ trough. The low frequency peak at 0.15 MHz, 3.2 mm/µs matches the intersection of A_0_ and A_1_ modes, and another peak at 0.65 MHz, 2.8 mm/µs also corresponds to the crossing of A_0_ and A_1_ modes. The asymptote of the A_0_ mode extends to 1 MHz in this plot, corresponding to the observed peak amplitude in the waveform at 106 ± 2 µs. Another spectrogram of the same signal showed the A_0_ mode asymptote extended to 2 MHz.

**Figure 6:**
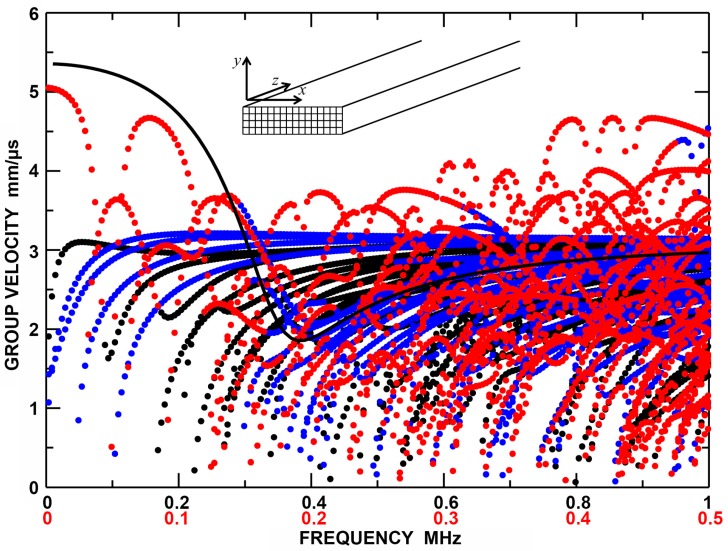
Dispersion curves of group velocity vs. frequency from semi-analytical finite-element [10]. **Red** points represent the modes dominated by z-direction displacement, **blue** points those by y-direction displacement and **black** points those by x-direction displacement. The group velocity of S_0_-mode plate wave for 6.4 mm-thick Al is drawn in as a black curve. Frequency scale for 12.7 mm thick bar is given in the second row (in red).

For bar waves generated using the 12.7 mm-thick bar, the waveform and CWT spectrograms are shown in Figure 5a,b. Again, Lamb wave dispersion curves for ten modes are overlaid to 800 kHz. Figure 5a is for the symmetrically excited case, corresponding to the plot for a 6.4 mm-thick bar given previously as Figure 4 in [10]. Only two peaks are common in both. The peak centered at 0.2 MHz, 1.7 mm/µs was marked cw1 peak in [10] and one at 0.15 MHz, 3.4 mm/µs was cw3. In Figure 5a, peaks corresponding to asymmetric modes are absent, as expected, and the strongest peak occurs at 0.43 MHz, 2.8 mm/µs. In terms of Lamb modes, this peak is at the intersection of S_0_ and S_1_ modes. According to the dispersion curves for this bar (Figure 6; a revised plot from [10]), several modes are present at that position. Since the bar cross section was proportionately doubled, the same dispersion curves can be used by halving the frequency scale. This is the lower scale (given in red) in Figure 6. Dispersion curves for aluminum and steel plates can be found readily in books on ultrasonic testing and those for steel are available in [19] up to the frequency-thickness product of 28 MHz·mm. Another peak at 0.18 MHz, 1.7 mm/µs occurs at the slower side of the S_0_ velocity trough. This slow-down is likely due to side-reflections in the bar. Thus, strong intensity peaks on a spectrogram can be interpreted well in terms of Lamb wave dispersion curves since the number of modes is high in bar-wave dispersion curves, making a proper selection difficult.

CWT result of asymmetric excitation for the 12.7 mm-thick bar is shown in Figure 5b. This spectrogram is completely different from Figure 5a for symmetric excitation. Strong part of waveform is also shorter than the symmetric case. The strongest peak occurred at 0.17 MHz and 3 mm/µs, where the bar-wave dispersion curves indicate the presence of four modes dominated by y-direction displacement (shown in blue curves in Figure 6). These modes appear to be from asymmetric modes. In terms of Lamb modes, this peak is at the intersection of A_0_ and A_1_ modes. Thus, Lamb wave dispersion curves again provide worthy guidelines for predicting bar wave propagation. In the velocity range of 2.7 to 3 mm/µs, moderate signal intensity persisted to 1 MHz. While a part of this activity may come from the SH mode, it is likely to be the result of the convergence of many modes dominated by y-direction displacement. A faint peak exists where a strong peak appeared in Figure 4a (at 0.2 MHz and 1.7 to 1.8 mm/µs), but most other peaks appear to be unrelated to symmetric Lamb mode peaks observed in Figure 4a.

### 3.2. Receiving Sensitivity to Guided Waves

In order to determine the receiving sensitivity of a sensor (or a transducer), it is necessary to mount it at the position where normal displacement has been characterized by steps described above. For each sensor, eight different sensitivity tests were conducted using four types of wave propagation media and two wave excitation methods. For the symmetric excitation with 12.7 mm-thick plate and 6.4 mm-thick bent bar set-ups, step pulses (367 V) were used for FC500 transducer, while mono-polar pulses of 329 V for FC500 transducer (and 298 V for V104 transducer that was used only for 6.4 mm-thick plate testing). The digitized sensor output was converted to the frequency domain using FFT as reported previously [9,10]. Subsequently, spectral division by already collected displacement data, also converted by FFT, yielded eight receiving sensitivities, one for each set-up. These were averaged and the averaged spectrum is taken as the receiving sensitivity of the sensor. Twelve transducers and sensors were used as receivers and listed in Table 1 along with KRN sensors and transmitters. Frequency listed is the nominal center frequency (in MHz) given by manufacturers.

### 3.3. Olympus V103 Transducer

**Figure 7:**
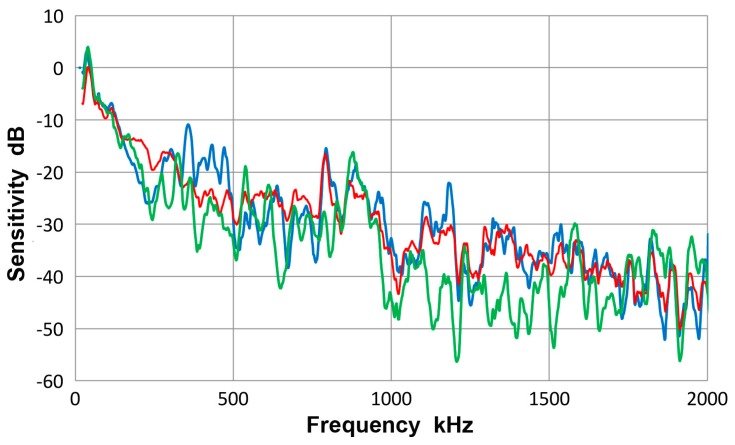
Receiving sensitivity of Olympus V103 transducer. **Red**-averaged guided wave sensitivity, **blue**-Lamb waves from symmetric excitation (S) for 12.7 mm plate, **green**-Lamb waves from asymmetric excitation (A) for the same plate.

This transducer is from Olympus NDT (Waltham, MA, USA), having a nominally 12.7 mm diameter, 1 MHz design for straight-beam ultrasonic applications. It is often used as a broadband AE sensor. Results of guided-wave displacement calibration tests are given in Figure 7 and in Appendix. Figure 7 shows three curves: blue is for symmetric excitation for 12.7 mm plate; green for asymmetric excitation for the same plate and red for the averaged spectrum from all eight tests. The vertical scale is in dB in reference to 0 dB at 1 V/nm. The two (blue and green) Lamb wave curves follow the same trend as the average (red) curve and the average difference between the two spectra was 4.1 dB. Locally, however, deviations were as large as 15 dB. The symmetric curve was mostly above the average. The general trend started with a sensitivity peak of 0 dB at 40 kHz. The sensitivity decreased with increasing frequency, dropping to −40 dB at 2 MHz. Similar tests were conducted using a thinner plate and two bars as wave propagation media and their results were comparable to Figure 7.

**Figure 8:**
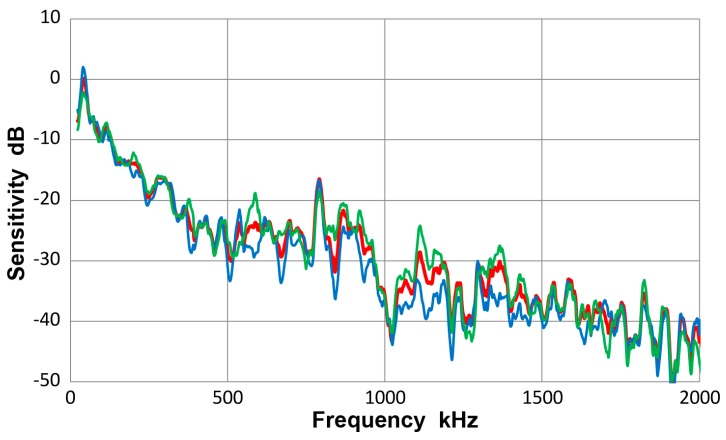
Receiving sensitivity of Olympus V103 transducer. **Red**-averaged guided wave sensitivity, **blue**-Lamb waves for 12.7 and 6.4 mm plates, **green**-bar waves for 12.7 and 6.4 mm bars.

**Figure 9:**
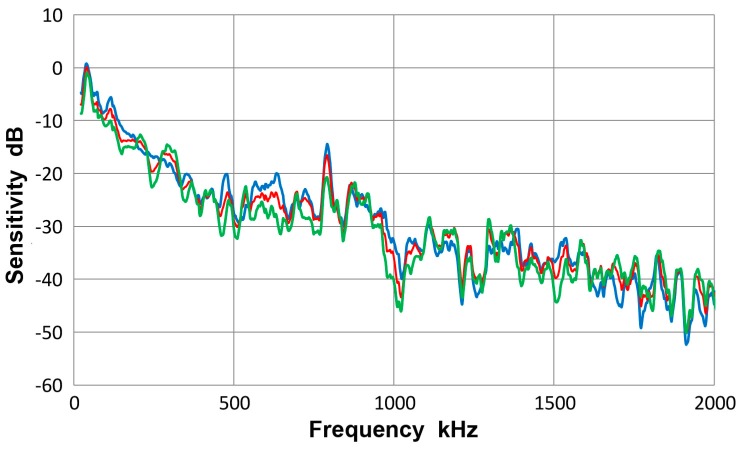
Receiving sensitivity of Olympus V103 transducer. **Red**-averaged guided wave sensitivity, **blue**-Lamb and bar waves from symmetric excitation (S) for 12.7 and 6.4 mm plates and bars, **green**-bar waves from asymmetric excitation (A) for the same four plates and bars.

Figure 8 gives a collection of three spectra with the average of eight curves in red, that of four Lamb-wave spectra in blue and that of four bar-wave spectra in green. Note that some of the data were from Appendix. These three curves agreed to each other closely, with the average difference of 0.36 dB from 22 kHz to 2 MHz. Observed agreement was better at lower frequency range; 0.14 dB to 0.5 MHz. The averages of standard deviation for the three averaged spectra were 5.2, 5.7 and 5.2 dB, so each of the average curves had considerable deviations. Figure 9 is similarly a collection of three spectra with the average of eight curves in red, that of four symmetric wave spectra in blue and that of four asymmetric wave spectra in green. Again, the mutual agreement among the three curves was good, with the average difference of 1.0 dB from 22 kHz to 2 MHz. The average spectral deviations were comparable at 5.2 and 5.5 dB for the symmetric and asymmetric waves.

Results illustrated in Figure 8 and Figure 9 indicate that the average curves match each other based on various grouping from different conditions exciting the sensor, although each average was deduced from data containing some deviations. This behavior leads to a conclusion that the averaged spectrum represents the receiving sensitivity of V103 sensor to guided waves. However, the presence of data scatter makes it necessary to use four (or more) different test set-ups for obtaining a measured sensitivity close to the average spectrum of the entire tests to within 1 dB.

In the following section, three averaged sensitivity curves for each sensor (the average of eight spectra, for Lamb waves and for bar waves) will be shown in order to present its observed receiving sensitivity to guided waves, as shown above in Figure 8 for V103. Results for four sensors (PAC HD50, S9220, µ30D and R15a) are presented in the Appendix.

### 3.4. PAC Pico Sensor

**Figure 10:**
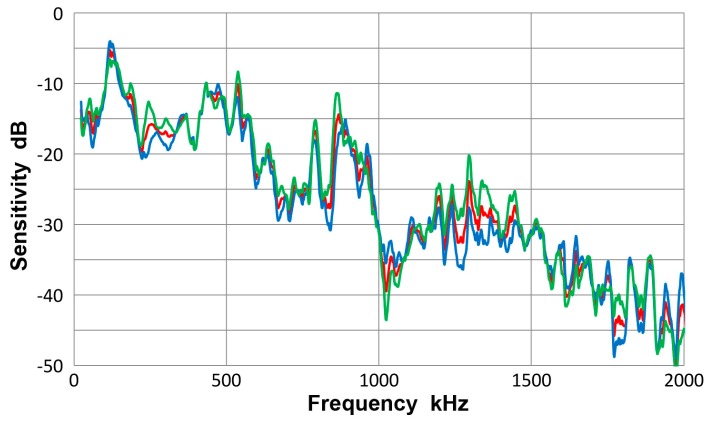
Receiving sensitivity of PAC Pico sensor. **Red**-averaged guided wave sensitivity, **blue**-Lamb waves for 12.7 and 6.4 mm plates, **green**-bar waves for 12.7 and 6.4 mm bars.

**Figure 11:**
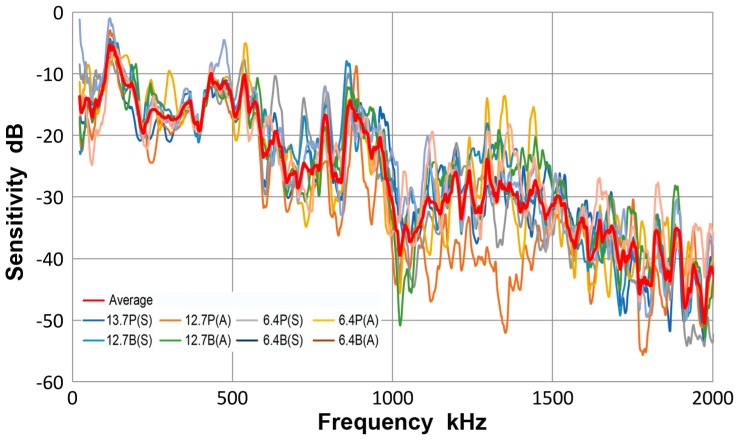
Receiving sensitivity of PAC Pico sensor. Average of eight curves-red, thick plate 12.7P(S)-blue, thick plate 12.7P(A)-orange, thin plate 6.4P(S)-grey, thin plate 6.4P(A)-yellow, thick bar 12.7B(S)- light blue, thick bar 12.7B(A)-green, thin bar 6.4B(S)-dark blue, thin bar 6.4B(A)-brown. Tests designated by thickness in mm, followed by P or B for plate or bar and (S) or (A) for symmetric or asymmetric excitation.

This sensor has an element of nominally 3.2 mm-diameter, 500 kHz design for broadband applications. It is from Physical Acoustics (PAC, Princeton Junction, NJ, USA). Three observed sensitivity spectra for the average of eight tests (red curve) and those for plates (blue curve) and for bars (green curve) are given in Figure 10. As in the case of V103 transducer, the three displacement sensitivity curves are practically identical, with the averaged standard deviation of 1.3 dB among the three spectra. That is, the results with plates and bars are close to each other. The overall scatter is shown in Figure 11, in which all eight spectra are plotted along with the averaged one (given in red curve). The averaged standard deviation was 3.9 dB to 2 MHz and to 1 MHz, it was down to 3 dB. This was less than the corresponding value of 5.2 dB for V103 (plots were not given). In this Pico sensor, it is concluded that a unique sensitivity exists for both Lamb waves and bar waves within the thickness range used.

The observed receiving sensitivity showed an abrupt drop at 1 MHz. For the frequency range above 0.5 MHz with thickness of a plate or a bar of 6.4 to 12.7 mm, the main wave propagation occurs at the velocity of approximately 3 mm/µs. Thus, the wavelength at 1 MHz corresponds to the sensor diameter of 3.2 mm. This produces displacement cancellation on the sensor surface, satisfying the null condition of aperture effect. When the frequency nears 0.5 MHz, the sensor diameter and the half-wavelength match and results in sensitivity maximum; in Figure 10, peaks are present between 430 to 540 kHz. Since this sensor also has thickness resonance mode at 500 kHz, it is also expected to produce a sensitivity maximum. Another sensitivity peak exists at low frequency (120 kHz in Pico) as in all other tests with Lamb and bar waves, but its origin is unclear at present.

### 3.5. PAC WD Sensor

**Figure 12:**
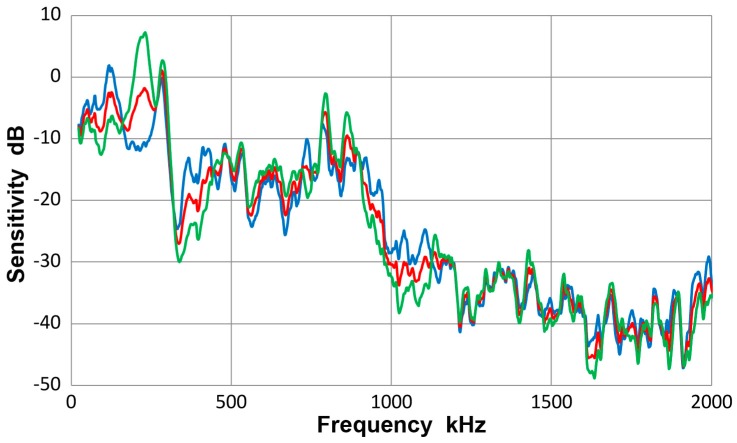
Receiving sensitivity of PAC WD sensor. **Red**-averaged guided wave sensitivity, **blue**-Lamb waves for 12.7 and 6.4 mm plates, **green**-bar waves for 12.7 and 6.4 mm bars.

This sensor is of multiple element construction in a housing for 12.7 mm-diameter sensors from PAC and has a non-flat broadband response. It is a convenient choice for detecting different frequency ranges at good sensitivity to NIW. Results for guided-wave sensitivity tests are shown in Figure 12. The averaged standard deviation among the three spectra was 2.1 dB to 2 MHz, but was higher at 3.5 dB for the range up to 1 MHz. Good sensitivity levels are found at 100 to 300 kHz range and at 800 kHz. At 300 kHz or below, three sensitivity spectra show significant spread, differing by as much as 18 dB. No unique guided-wave sensitivity is present. Above 0.5 MHz, however, the averaged standard deviation is 1.7 dB, almost as low as the smaller sensor group. Because of a large drop-off at 1 MHz, higher frequency use is not feasible for WD.

In WD sensor, the sensitivities at 225 kHz are difficult to understand as it dips for Lamb-wave reception, but peaks for bar-wave receiving sensitivity. It also showed an opposite effect at 1 to 1.2 MHz.

### 3.6. DECI SH225 Sensor

**Figure 13:**
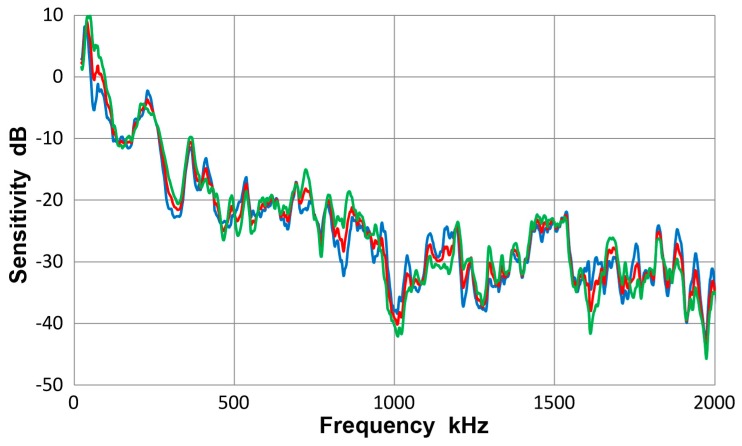
Receiving sensitivity of DECI SH225 sensor. **Red**-averaged guided wave sensitivity, **blue**-Lamb waves for 12.7 and 6.4 mm plates, **green**-bar waves for 12.7 and 6.4 mm bars.

This shear-wave sensor was developed at Dunegan Engineering (DECI, now Score Atlanta, Kennesaw, GA, USA). It is designated to have 225 kHz-center frequency for shear waves using a rectangular element. The shear motion is along the short direction. In the present testing, guided waves were received in the same way, that is, the wave direction coincided the short direction. Results are given in Figure 13. The averaged standard deviation among the three spectra was 1.4 dB to 2 MHz, matching the lowest value found above. Displacement sensitivity was the highest at 40 kHz and also peaked at 225 kHz and at 360 kHz. Reflecting the large size of the sensing element, it has good receiving sensitivity, reaching 10 dB. The peak at 225 kHz was perhaps designed through the size of the rectangular piezoelectric element of 6.3 mm. For typical Rayleigh-wave velocity of 2.9 to 3.2 mm/µs for steel and aluminum alloys, this corresponds to half the wavelength. The same principle leads to maximizing the guided wave sensitivity as observed in this test. However, observed sensitivity dips at 360 kHz and 460 kHz and a peak at 540 kHz are difficult to reconcile with the aperture effect interpretation.

### 3.7. PAC R6a Sensor

**Figure 14:**
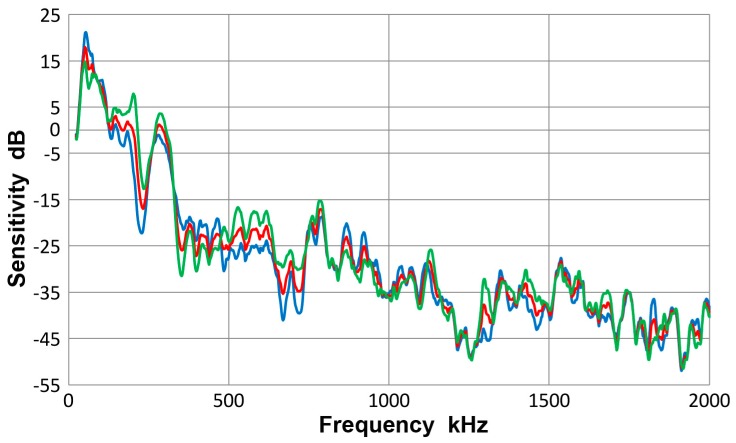
Receiving sensitivity of PAC R6a sensor. **Red**-averaged guided wave sensitivity, **blue**-Lamb waves for 12.7 and 6.4 mm plates, **green**-bar waves for 12.7 and 6.4 mm bars.

This sensor is a nominally 12.7 mm-diameter design for low frequency applications and is intended for Rayleigh-wave detection. Figure 14 provides the results of sensitivity determination using plates and bars. The averaged standard deviation among the three spectra was 1.9 dB to 2 MHz, only 0.5 dB higher than the lowest value found above. The sensitivity was the highest at 50 kHz, reaching 17 dB, the highest value among the sensors tested in this study. After a peak at 280 kHz, it dropped 20 dB, followed by a slow decrease in sensitivity to 2 MHz. A sensitivity dip is found at 230 kHz. This dip was hardly observed in the previous bar-wave test with 6.4 mm-thick bar [10]. In this study, the thicker bar and plate tests contributed strongly to this dip, and made this a prominent feature. For this sensor size, wave velocity of 2.4 mm/µs gives the first order null at 230 kHz. This sensor is expected to have a more complex sensing element than a round disc, but the aperture null effect does explain the observed 230-kHz dip.

**Figure 15:**
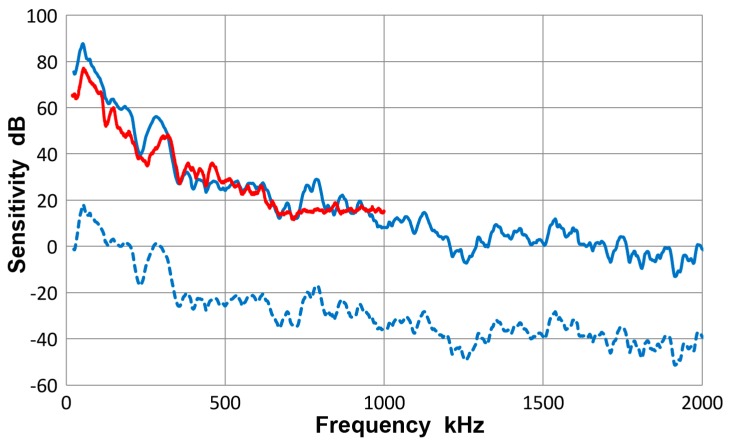
Receiving sensitivity of PAC R6a sensor. **Blue dash**-averaged guided wave displacement sensitivity, **blue**-Velocity response of the averaged curve, **red**-surface wave velocity response supplied by PAC.

For this sensor, PAC supplied a Rayleigh-wave velocity calibration. It is compared to the average guided-wave receiving sensitivity in Figure 15. The PAC calibration is in red curve, while the guided-wave receiving sensitivity (converted to velocity sensitivity by multiplying with the angular frequency) is shown in blue. The corresponding displacement sensitivity is given in blue dash curve. The peak value of the Rayleigh-wave velocity sensitivity was 77.1 dB and was 10 dB lower than the guided-wave velocity sensitivity, as noted previously in the report of the bar-wave test using 6.4 mm-thick bar [10]. However, the present guided-wave sensitivities decreased over 100 to 200 kHz range, improving the agreement further. PAC’s Rayleigh-wave velocity calibration also confirms the presence of a sensitivity dip near 250 kHz.

### 3.8. PAC R15 Sensor

**Figure 16:**
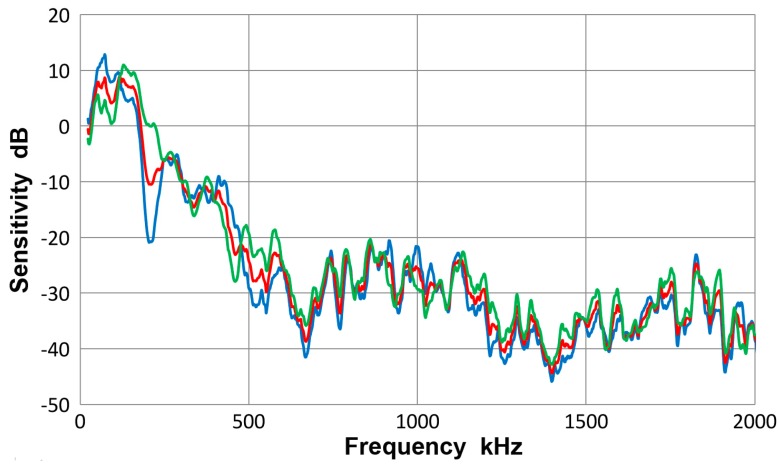
Receiving sensitivity of PAC R15 sensor. **Red**-averaged guided wave sensitivity, **blue**-Lamb waves for 12.7 and 6.4 mm plates, **green**-bar waves for 12.7 and 6.4 mm bars.

This sensor is a nominally 12.7 mm-diameter design for general AE applications. It has the main sensitivity peak nominally at 150 kHz. This and similarly designed sensors, which used piezoelectric ceramic discs of 12.7 mm diameter and 6.4 mm thickness, have been widely used since the 1960s. Figure 16 shows the results of sensitivity determination using plates and bars. The averaged standard deviation among the three spectra was 2.0 dB to 2 MHz, similar to that of R6a sensor above. The sensitivity was the highest at 125 kHz, reaching 8.5 dB. It dropped beyond 180 kHz, followed by a sensitivity dip at 200 kHz. As was discussed for R6a sensor above, this dip was not observed in the previous bar-wave test with 6.4 mm-thick bar [10]. Here, the thicker bar and plate tests contributed strongly to this dip. For this sensor size, wave velocity of 2.0 mm/µs gives the first-order null at 190 kHz. For this frequency and wave velocity, the S_0_ and S_1_ modes contribute to a strong intensity peak for the 12.7 mm-thick plate and bar (see Figure 4a and Figure 5a). Thus, the dip appears to originate from sensor response to one of the thicknesses used. This points to a caution needed for averaging sensitivity results from different wave propagation media. Overall, however, the averaging provides the benefit against data scatter. Three higher order nulls are predicted at 349, 506 and 663 kHz using the same parameters. These are detected in Figure 16 at 345, 511 and 668 kHz, matching the predictions well.

### 3.9. PAC F30a Sensor

**Figure 17:**
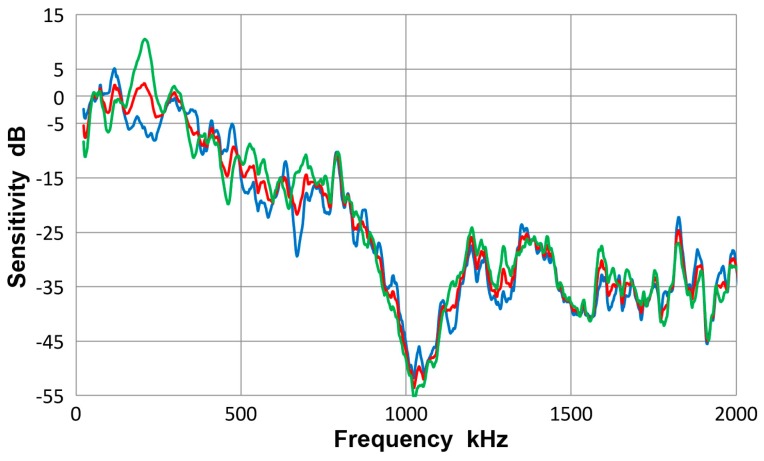
Receiving sensitivity of PAC F30a sensor. **Red**-averaged guided wave sensitivity, **blue**-Lamb waves for 12.7 and 6.4 mm plates, **green**-bar waves for 12.7 and 6.4 mm bars.

This PAC sensor exhibits a flat response in receiving sensitivity to NIW [10], based on a multiple-sensing-element design. Its sensitivities to Lamb waves and bar waves are shown in Figure 17. Relatively good sensitivity was found to 300 kHz. A steep decrease in receiving sensitivity with increasing frequency observed in many other sensors in this study is absent until frequency reached 0.8 MHz with a dip at 1 MHz. The averaged standard deviation among the three spectra was 1.9 dB to 2 MHz. This average value is misleading since large discrepancies exist below 250 kHz between Lamb- and bar-wave sensitivities. Previously, a similar behavior was seen for WD sensor and, to a lesser extent, in both R15 and R15a sensors. Their cause is not known at present.

In spite of the different responses to Lamb and bar waves, this F30a sensor is notable for a broader frequency ranges at low frequencies. For many practical applications, this provides an improved detection capability of AE events.

### 3.10. Olympus V101 Transducer

**Figure 18:**
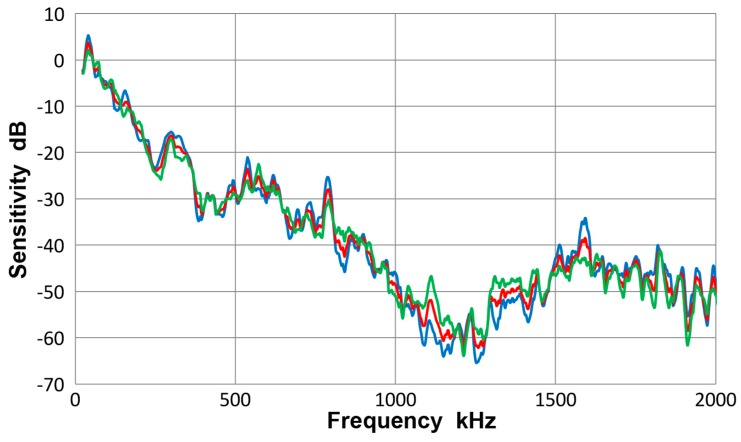
Receiving sensitivity Olympus V101 transducer. **Red**-averaged guided wave sensitivity, **blue**-Lamb waves for 12.7 and 6.4 mm plates, **green**-bar waves for 12.7 and 6.4 mm bars.

This is a broadband 0.5 MHz ultrasonic transducer and is an unlikely candidate for use as an AE sensor unless low frequency broadband sensitivity is required. It was selected as an example of a large aperture size (25.4 mm), which is expected to reduce higher frequency sensitivity to guided waves. Because of its large size, chances of waveform cancellation are larger. Results are given in Figure 18. Indeed, sensitivity diminished at higher frequencies, confirming the expected behavior for a large aperture sensor. The averaged standard deviation among the three spectra was 1.5 dB giving good agreement among different wave types. The peak at 40 kHz is high, but the sensitivity decreased with increasing frequency. The dip at 1.2 MHz is due to the anti-resonance from its fundamental response. Dips at 350 and 390 to 440 kHz can be the second and third order null frequencies, taking the wave velocity as 2.8 mm/µs.

### 3.11. Summary of Guided Wave Sensitivity

(a) On the basis of the bar-wave receiving sensitivities of point-contact KRN sensors, guided-wave receiving sensitivities of 12 ultrasonic transducers and AE sensors were determined. For each transducer or sensor, a unique sensitivity emerged after averaging eight different experimental spectra. Eight test conditions were formed from two geometries of propagation media, plate and bar, their thickness, 12.7 and 6.4 mm and the mode of guided wave excitation, symmetric and asymmetric.
(b) The guided-wave receiving sensitivities thus determined were slightly different from the bar-wave sensitivities reported previously for the same transducers and sensors, which relied on a single test condition (bar wave, 6.4 mm thickness, symmetric excitation). At least four different conditions are needed to cancel out various effects of test conditions that arise from different wave mode excitation and propagation and from aperture null effect dependent on wave velocity and sensing element size.
(c) The guided-wave receiving sensitivities obtained were completely different from the receiving sensitivities to NIW. This conclusion was identical to that reported for the cases when single bar-wave testing set-up was utilized [10].
(d) The guided-wave receiving sensitivities showed a single intense peak or multiple intense peaks at low frequencies, typically below 300 kHz. The sensitivities always decreased with increasing frequencies. When the sensing element size is small (e.g., Pico, HD50 and S9220), or with multiple element construction (WD), a few more higher frequency peaks were observed in addition at around 0.5 and/or 0.8 MHz. In one case (F30a), three peaks appeared below 0.3 MHz, providing a broader frequency response.

### 3.12. Frequency Dependence of Receiving Sensitivity

**Figure 19:**
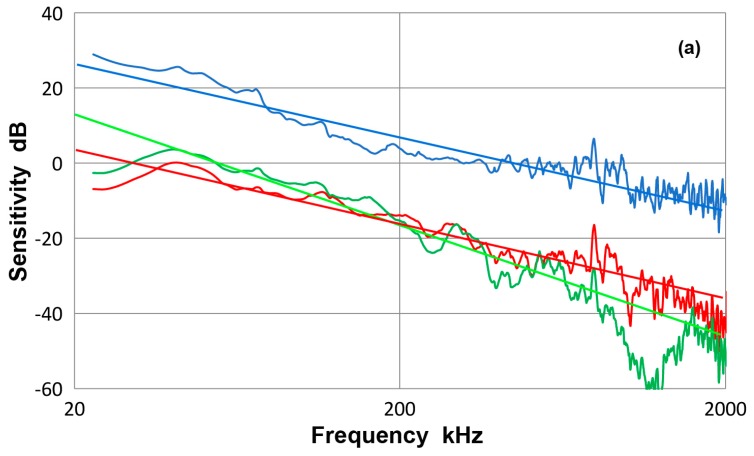

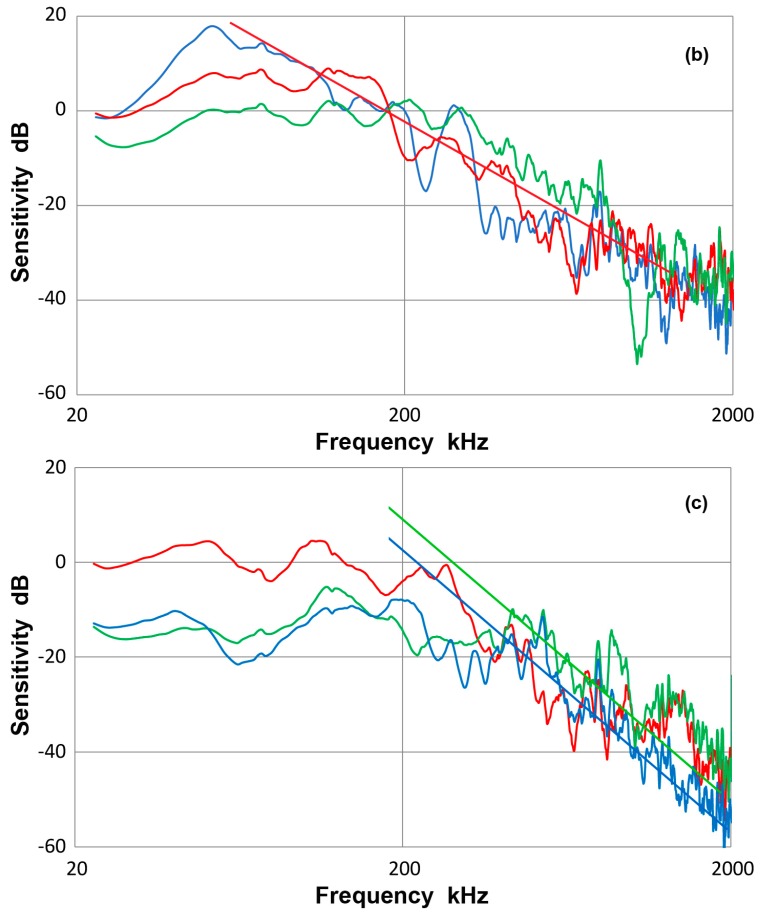
(**a**) Frequency dependence of guided wave sensitivity. **Blue**-KRN sensor with FET buffer (#11053), red-V103 transducer, **green**-V101 transducer. **Blue** and **red** lines: slope = 1, **Green** line: slope = 1.5. (**b**) Frequency dependence of guided wave sensitivity. Blue-R6a sensor, **red**-R15 sensor, **green**-F30a sensor. **Red** line: slope = 2. (**c**) Frequency dependence of guided wave sensitivity. **Blue**-HD50 sensor, **red**-µ30D sensor, **green**-Pico sensor. **Blue** and **green** lines: slope = 3.

All the receiving sensitivity curves obtained in this study decreased with frequency, varying smoothly over some ranges at least. Many of the observed sensitivity curves exhibit power-law behavior, which may indicate underlying physical mechanisms. These for KRN sensors and Olympus transducers (shown earlier in Figure 1, Figure 8 and Figure 18) are replotted in Figure 19a with the logarithmic frequency scale. With sensitivity in dB, this is a log-log plot and a linear slope represents a power-law exponent. KRN sensor and V103 can be fitted approximately to a 1/*f* dependence as represented by blue and red lines. Here, *f* is frequency. V101 transducer gives a larger slope of −1.5 or 1/f^1.5^ dependence. Another group of PAC sensors gives a steeper frequency dependence of 1/*f*^2^ in the frequency range above their respective resonance, as shown in Figure 19b. A group of small sensors, PAC HD50, µ30 and Pico sensors, showed another characteristic behavior. This is shown in Figure 19c. Past their resonance, the steepest slope of −3 is found, or 1/*f*^3^ dependence. Of the observed frequency dependence, the 1/*f* dependence can be considered to correspond to a constant velocity response of the receiving sensitivity. In the present cases for KRN and V103, the flat velocity range extends over nearly two decades. This behavior is difficult to attribute to a simple model of coupled resonator, often invoked to describe resonant AE sensors [26]. With this model, the velocity response is predicted at the resonance region and is inapplicable to the observed behavior in Figure 19a, where 1/*f* dependence covers more than a decade of frequency. The case of 1/*f*^2^ dependence is similarly considered in terms of acceleration response, but again this cannot be explained by any plausible mechanical model. Other frequency dependence with the slope of −1.5 or −3 cannot be attributed to any conceivable model as well. Thus, it is necessary to leave the interpretation of the observed frequency dependence open for future work.

### 3.13. Asymmetric Excitation

**Figure 20:**
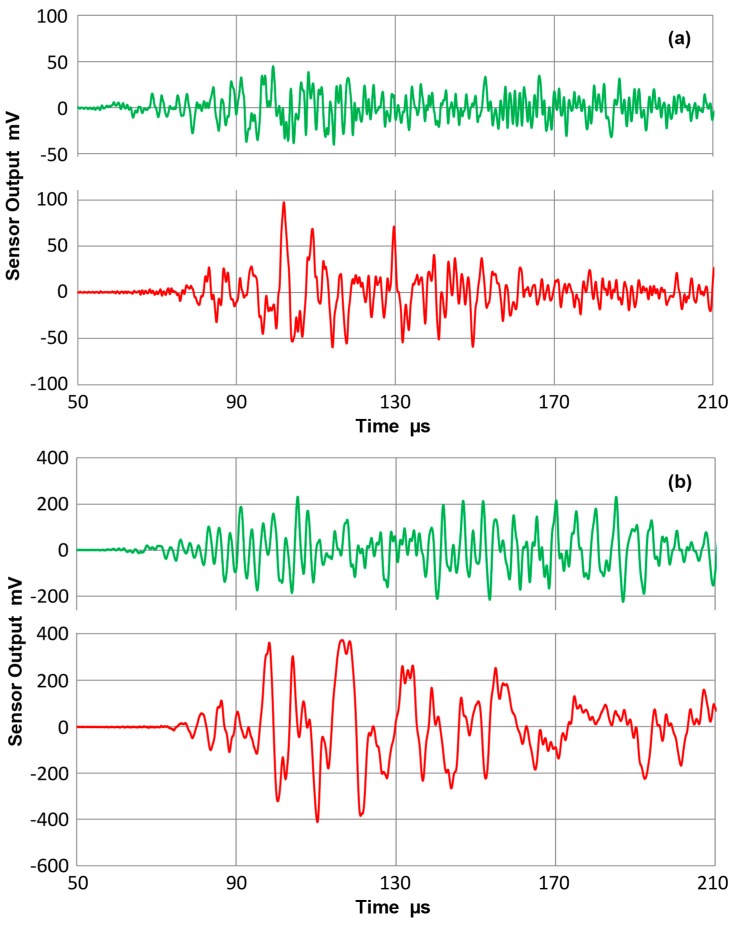
Waveforms of the sum (**green** curve) and difference (**red** curve) of bar waves from two surfaces. 12.7 mm-thick bar at 300 mm distance. (**a**) Asymmetric excitation from an end face using FC500 transmitter. (**b**) Asymmetric excitation from an end face using a shear transmitter (SH225).

**Figure 21:**
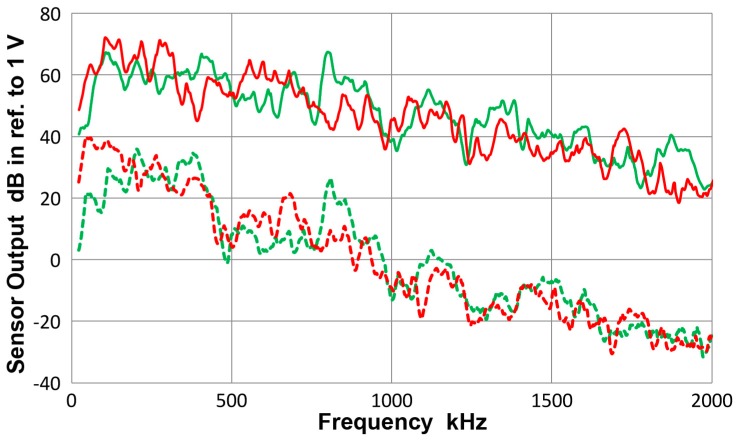
Frequency spectra of the four waveforms in Figure 20. **Green** curves for the sum and **red** for the difference. Solid curves are for FC500 transmitter and dashed ones for SH225 transmitter, which were shifted down 50 dB for better visibility.

During the testing with asymmetric excitation, some symmetric components were also observed. By detecting displacement responses on both sides on a bar, the extent of symmetric mode contribution was examined. Figure 20a gives the sensor outputs on the 12.7 mm-thick bar after taking the sum and difference of outputs from both surfaces, front and back. The top curve (green) represents the symmetric component and the bottom curve (red) the asymmetric component. The latter is approximately twice over time period of 100 to 150 µs. Figure 20b shows results from another method of asymmetric excitation, in which a shear sensor (DECI SH225) was glued to the bar end. The peak output was four time larger since the contact area is about 1/3 larger and the vibration mode is in shear. However, the waveforms indicate lower frequency features predominantly. The frequency spectra of these four waveforms are compared in Figure 21. The solid curves are for the standard method used and the dashed curves utilized the shear transmitter (SH225). The latter curves were shifted down 50 dB to separate the plots. In both types of excitation, asymmetric spectra are stronger at low frequencies, up to 300 kHz for the solid curve and to 200 kHz for the dashed curve. The spectral intensities of some regions had symmetric modes above asymmetric modes, especially above 800 kHz. The shear transmitter appears to suffer from the aperture null effect even for transmission as its output had a dip at 0.5 MHz, where the shear wavelength matches its active element width of 6.3 mm. When the need for sensor characterization is limited to below 0.5 MHz, the use of this shear transmitter is worth considering since it can be securely bonded to the bar end.

### 3.14. Thickness Effects

**Figure 22:**
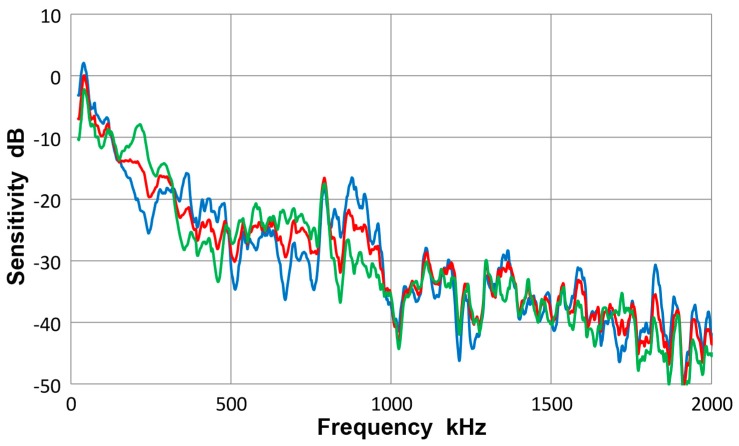
Receiving sensitivity of Olympus V103 transducer. **Red**-averaged guided wave sensitivity, **blue**-Lamb and bar waves from 12.7 mm-thick plate and bar, **green**-Lamb and bar waves from 6.4 mm-thick plate and bar.

When the receiving sensitivity data for V103 transducer is grouped by the thickness, results are given in Figure 22. Blue curve represents 12.7 mm-thick plates and bars and green curve 6.4 mm-thick plates and bars. The average of all the spectra is again given by the red curve. The average spectral difference was 0.6 dB, as noted before in Section 3.3, but the maximum difference reached almost 14 dB. The observed differences in the sensitivity spectra chiefly arose from the aperture null effect. For the two thicknesses, the null frequencies observed and their ratios are in Table 2: 

The ratio of each pair is close to the thickness ratio of 2.0. Because of this major aperture effect, it is inappropriate to rely on a single thickness in order to deduce the receiving sensitivity of a sensor. By averaging data of different conditions, it is possible to approximate the sensor’s own response.

In practical AE applications involving structures of a certain thickness, on the other hand, it is proper to consider the responses of AE sensors for that thickness. This will most likely require users to set up own calibration facility as manufacturers will be unable to accommodate numerous possibilities. This practice is common in some ASME and ASTM standard procedures [13,14]. The set-up needs not require the absolute calibration of displacement or velocity. Sensors can be validated in a relative scale as it has been conducted in the past with AE sensors for NIW and for composite testing.

### 3.15. Recommendations

In many applications of AE, acousto-ultrasonics and structural health monitoring, it is necessary to detect guided waves for achieving successful materials evaluation. It is essential for such endeavors to recognize that guided wave sensing requires a strategy different from that for NIW. Receiving sensitivities for these two types of ultrasonic waves are distinct and their calibration must be conducted accordingly.

(a) When one requires calibration in absolute units, use a laser interferometer and obtain the displacement spectrum on a long bar (>1 m length and a thickness-to-width ratio of 1:4 recommended), mounting longitudinal and shear wave transmitters on both ends for generating symmetric and asymmetric bar waves. Select another bar of different size, also mount longitudinal and shear transmitters on both ends, giving four set-ups. Determine the receiving sensitivities for the four different set-ups and average the results as conducted in the present work. For further improvement, include more thickness values.
(b) For practical standardization of the sensor sensitivity to guided waves, one bar (>1 m length as above) can be used with one longitudinal wave transmitter on one end, obtaining a displacement (or velocity) output spectrum with a laser interferometer (or vibrometer) at a designated sensing position. A calibration spectrum is supplied in terms of excitation voltage-referenced displacement (or velocity). A recent work [29] showed that nearly identical sensitivity results were obtained from using a single-point laser interferometry and an averaged scanning laser vibrometry (for NIW). Thus, it is likely that the choice of laser instruments is not critical. While absolute calibration cannot be made, when the bar size is controlled, a guided wave sensitivity is obtained and can be compared among similarly calibrated sensors. This allows repeatable field measurements of sensor sensitivity to guided waves.
(c) When only performance validation is required, skip the laser measurement, use one bar, still use both symmetric and asymmetric bar waves, obtain a reference sensor with known guided-wave sensitivity and get relative performance of sensor-under-test by comparing to the reference.
(d) In guided-wave sensing, one must realize that useable frequency range is severely limited for typical AE sensors and ultrasonic transducers. Reducing the size helps, but the sensitivity is reduced. Newer composite-element sensor designs are needed to increase the performance at higher frequency.

Finally, guided-wave sensitivity revealed in this study resolves some of puzzling questions from past AE results. Two examples are given: one is on tensile testing of a magnesium alloy that used correlation analysis for deducing the frequency spectra from random signals [30]. Using Dunegan (now DECI) S140B sensor, the initial frequency spectrum at yielding had two peaks at 80 and 160 kHz. As work-hardening started, the peak at 80 kHz dropped 15 dB. (In retrospect, this is due to a shift in deformation mechanism from twinning to dislocation glide). The same S140B sensor from the old study (almost 50 years since its manufacture), like R15 considered in Section 3.8, had diminishing low-frequency sensitivity to NIW. The S140B sensor was also tested for bar-wave sensitivity, which showed a nearly identical shape as the green curve in Figure 16. The peak level was, however, lower by 5 dB for the primary peaks (110 to 160 kHz) and by 10 dB for the secondary peak at 55 to 75 kHz. The guided wave sensitivity below 80 kHz thus detected twinning deformation. Another puzzle was found when using WD sensor (see Section 3.5). In normal sensor calibration, WD has good response above 200 kHz, peaking around 500 kHz [10]. In composite sheet tensile testing, Huang saw peaks centering at 95 or 30 kHz [31,32]. The guided wave sensitivity for WD (Figure 12) has good responses below 300 kHz and again explains Huang’s observations. Many past test results were interpreted without accounting for guided wave responses. This will have to be remedied in future AE studies.

## 4. Conclusions

(a) The characteristics of piezoelectric detectors, or ultrasonic transducers and acoustic emission sensors, for quantifying the wave motion of guided ultrasonic waves, have been evaluated systematically. This study relied on laser interferometry for the base displacement measurement of bar waves, and determined surface displacements of eight different guided-wave test set-ups. These were used to obtain guided-wave receiving sensitivities of 12 ultrasonic transducers and AE sensors. Both plates and bars of 12.7 and 6.4 mm thickness were used as wave propagation media. These were excited with pulse-driven ultrasonic transmitters in symmetric and asymmetric manner. The upper frequency limit was 2 MHz.
(b) Generally, the receiving sensitivities showed rapidly dropping response with increasing frequency due to waveform cancellation on their sensing areas. This effect contributed to vastly different sensitivities to guided waves and to normally incident waves.
(c) The guided-wave receiving sensitivities showed a single intense peak or multiple intense peaks at low frequencies, typically below 300 kHz. The sensitivities always decreased with increasing frequencies. When the sensing element size is small (e.g., Pico, HD50 and S9220), or with multiple element construction (WD), a few more higher frequency peaks were observed in addition at around 0.5 and/or 0.8 MHz. In one case (F30a), three peaks appeared below 0.3 MHz, providing a broader frequency response.
(d) Various other effects are discussed and recommendations on methods of implementing the approach developed here are provided.

## Figures and Tables

**Table 1:** Transducers and sensors used.

Transducer Model	Manufacturer	Frequency MHz	Element Size mm
FC500	AET Corp	2.25	19 ^T^
V104	Olympus	2.25	25 ^T^
KRNBB-PCP or -PC	KRN Services	0.1–1	1
V103	Olympus	1	12.7
V101	Olympus	0.5	25.4
R6-alpha	Physical Acoustics	0.06	12.7
R15	Physical Acoustics	0.15	12.7
R15-alpha	Physical Acoustics	0.15	12.7
F30-alpha	Physical Acoustics	0.2–0.7	12.7 *
WD	Physical Acoustics	0.3–0.5	12.7 *
HD-50	Physical Acoustics	0.5	3
µ30D	Physical Acoustics	0.3	8
Pico	Physical Acoustics	0.5	3.2
S9220	Physical Acoustics	0.9	8
SH-225	Dunegan Engineering	0.225	6.3 × 12.6 **

^T^ Transmitter; * Multiple elements; ** Rectangular shear element.

**Table 2:** Frequency of aperture null effects and null frequency ratio.

Sensor Diameter	1	2	3	4	5	
12.7 mm	260	515	670	760	1024	(kHz)
6.4 mm	454	1024	1213	1565	2058	(kHz)
Frequency Ratio	1.75	1.99	1.81	2.06	2.02.	

**Figure A1:**
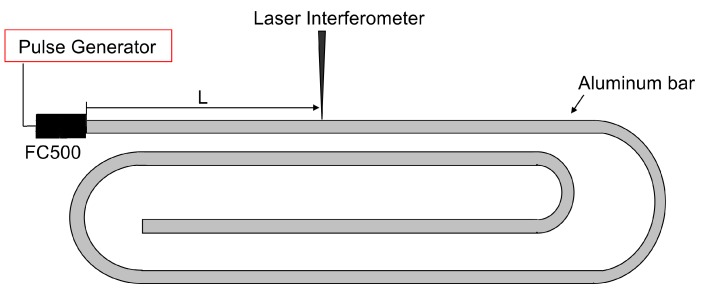
A bar-wave calibration set-up [10]. Bar size was 6.4 mm × 25.4 mm × 3600 mm.

**Figure A2:**
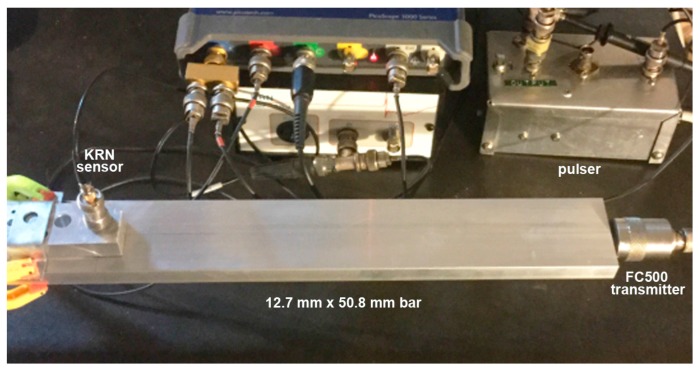
Photographs of a bar-wave calibration set-up. Bar size was 12.7 mm × 50.8 mm × 1220 mm. AET FC500 transmitter is glued on end face and a KRN sensor is positioned at 300 mm.

**Figure A3:**
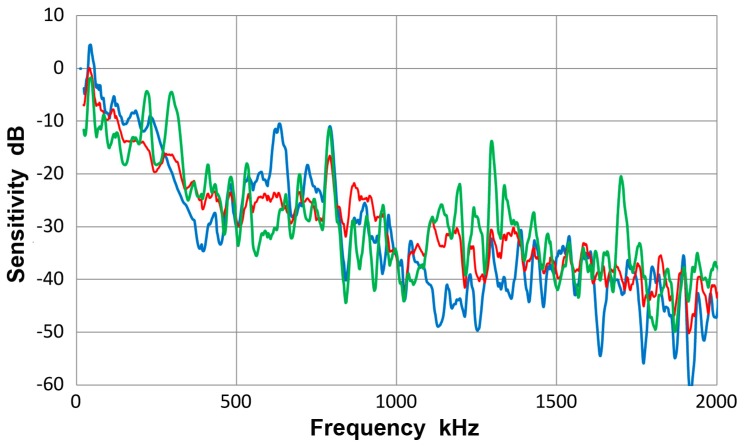
Receiving sensitivity of Olympus V103 transducer. **Red**-averaged guided wave sensitivity, **blue**-Lamb waves from symmetric excitation for 6.4 mm plate, **green**-Lamb waves from asymmetric excitation for the same plate.

**Figure A4:**
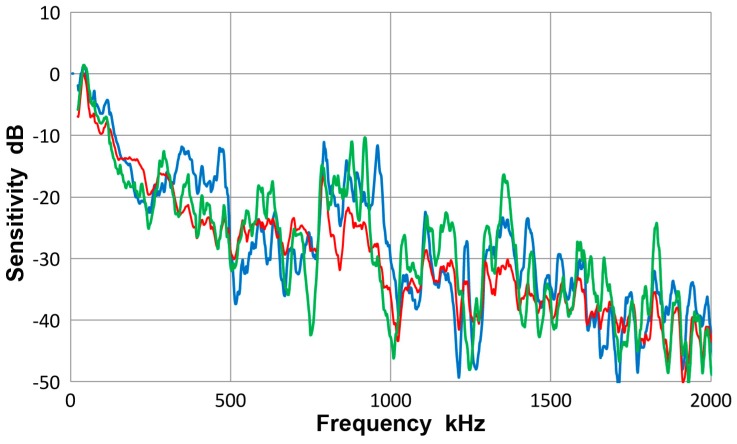
Receiving sensitivity of Olympus V103 transducer. **Red**-averaged guided wave sensitivity, **blue**-bar waves from symmetric excitation for 12.7 mm bar, **green**-bar waves from asymmetric excitation for the same bar.

**Figure A5:**
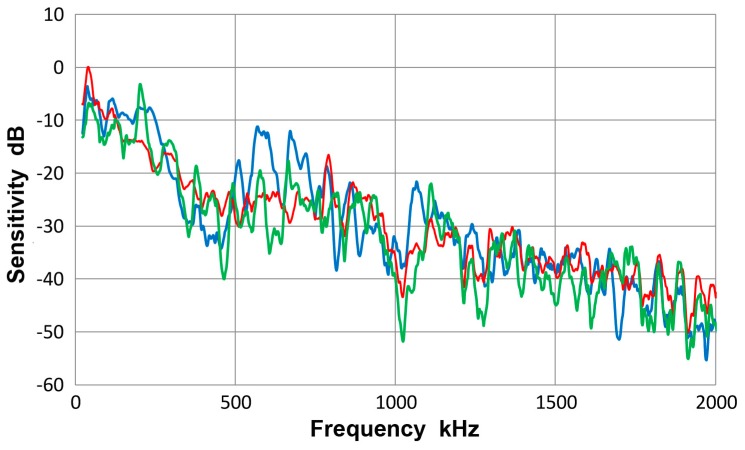
Receiving sensitivity of Olympus V103 transducer. **Red**-averaged guided wave sensitivity, **blue**-bar waves from symmetric excitation for 6.4 mm bar, **green**-bar waves from asymmetric excitation for the same bar.

**Figure A6:**
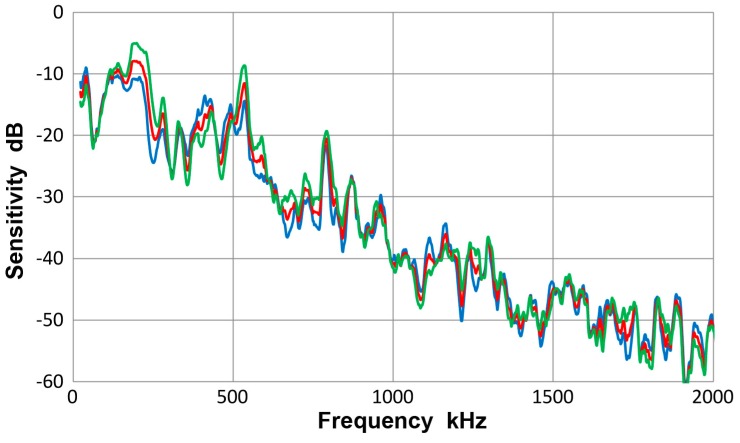
Receiving sensitivity of PAC HD50 sensor. **Red**-averaged guided wave sensitivity, **blue**-Lamb waves for 12.7 and 6.4 mm plates, **green**-bar waves for 12.7 and 6.4 mm bars.

**Figure A7:**
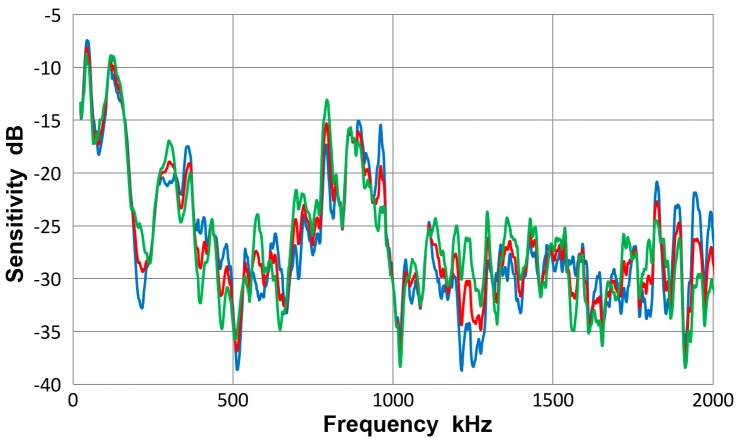
Receiving sensitivity of PAC S9220 sensor. **Red**-averaged guided wave sensitivity, **blue**-Lamb waves for 12.7 and 6.4 mm plates, **green**-bar waves for 12.7 and 6.4 mm bars.

**Figure A8:**
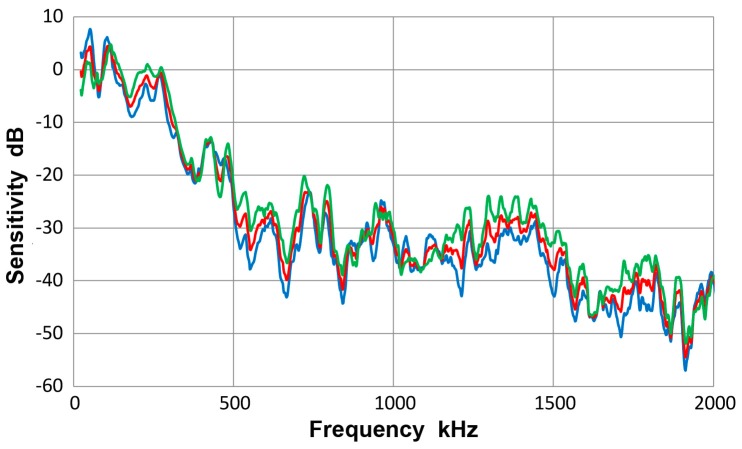
Receiving sensitivity of PAC µ30D sensor. **Red**-averaged guided wave sensitivity, **blue**-Lamb waves for 12.7 and 6.4 mm plates, **green**-bar waves for 12.7 and 6.4 mm bars.

**Figure A9:**
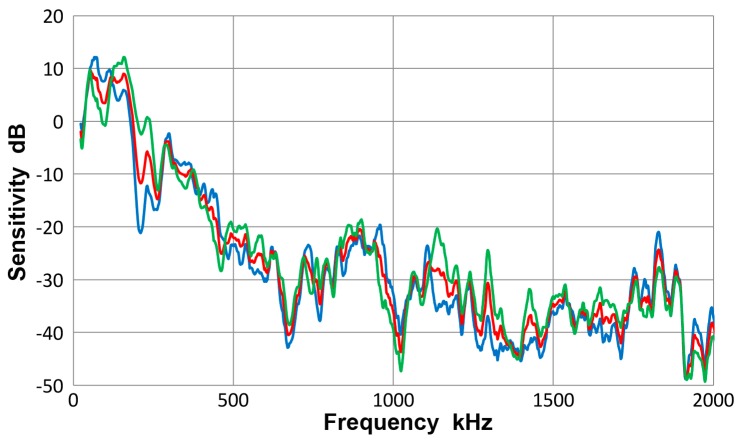
Receiving sensitivity of PAC R15a sensor. **Red**-averaged guided wave sensitivity, **blue**-Lamb waves for 12.7 and 6.4 mm plates, **green**-bar waves for 12.7 and 6.4 mm bars.

## Appendix A

### A.1. Calibration Set-Ups

A bar-wave calibration set-up is shown in Figure A1 [10]. A transmitter is glued to one end of a long, bent bar (>3 m long) with an initial straight section (700 mm long). The main sensing location is at L = 300 mm from the transmitter. The displacement of this set-up was characterized using laser interferometry and used as the basis of the present sensor calibration study. Figure A2 is a shorter bar (~1 m long) with longitudinal or shear wave transmitter. Again, the sensing position is at 300 mm from the transmitter. During measurement with a sensor at the sensing location, the 300 mm-section remains free, but there was no change in detected waveforms even when a small polymer support is inserted.

### A.2. Spectral Variation from Guided-Wave Variables

Spetral output from V103 transducer was converted to its bar-wave sensitivity by subtracting the known displacement spectrum at the sensing location. Two of them were shown in Figure 7 earlier in Section 3.3. There are six more different combinations of wave excitation modes, plate or bar propagation medium, and two thicknesses. Three pairs are shown below. Figure A3 compares the average with two cases for 6.4 mm-thick plate. Three curves have the same color code as in Figure 7. That is, blue is for symmetric excitation for 6.4 mm plate; green for asymmetric excitation for the same plate and red for the averaged spectrum from all eight tests. Here, the trend repeated as in the 12.7 mm-plate cases, except the asymmetric (green) curve was mostly higher than the average. The average spectral difference was 4.3 dB. In both plate cases, results deviated from the average widely, but the average curve tended to follow the middle point of the two Lamb wave sensitivities.

Next, bar-wave results are examined. Figure A4 shows the receiving sensitivities to bar waves with 12.7 mm-thick bar as the wave propagation medium. Again, three curves are plotted; blue is for symmetric excitation for 12.7 mm bar; green for asymmetric excitation for the same bar and red for the averaged spectrum from all eight tests. Figure A5 is the same plot for 6.4 mm-thick bar. Here, the deviation from the average is slightly reduced from those found in the plate cases of Figure 7 and Figure A3. The average spectral difference was 3.5 dB for both Figure A4 and Figure A5. All the curves in both figures are within a 10 dB-wide band up to 0.4 MHz. The spectral difference for the bar waves is slightly lower, but deviation from the average tended to be larger than the Lamb wave cases. However, the overall trend followed that of Lamb-wave sensitivities. Another grouping approach is to separate data according to the thickness of plates and bars. It is obvious, however, that aperture null effects produce different sensitivity spectra and this was considered in Section 3.14. Note that the average spectral difference was 0.6 dB, smaller than any of the four groupings discussed above.

### A.3. Sensor Characteristics

#### A.3.1. PAC HD50 Sensor

This sensor has an element of nominally 3 mm-diameter at 500 kHz for stud-mount applications from PAC. Three observed sensitivity spectra for the average of eight tests (red curve) and those for plates (blue curve) and for bars (green curve) are given in Figure A6. In resemblance to the case of a small Pico sensor, the three displacement sensitivity curves are practically identical, with the averaged standard deviation of 1.4 dB among the three spectra. This observation shows that the Lamb- and bar-wave results are close to each other. The overall scatter was also reduced (plots omitted) in comparison to V103, but the averaged standard deviation for the eight spectra was 4.1 dB to 2 MHz, higher than Pico discussed above. In HD50, it can again be concluded that a unique receiving sensitivity exists for both Lamb waves and bar waves within the thickness range used.

The sensitivity plots again point to a peak near 0.5 MHz and show a gradual drop with increasing frequency. Again, a prominent low-frequency peak exists at 0.2 MHz.

#### A.3.2. PAC S9220 Sensor

This sensor, also from PAC, is nominal 900 kHz design with a piezoelectric element of 8 mm in diameter. Results of receiving sensitivity tests are summarized in Figure A7. This is a comparable plot to Figure 12 and Figure 14, but the vertical scale is expanded twice, giving the appearance of larger scatters. However, the averaged standard deviation among the three spectra was 1.4 dB as in Figure 10. Its sensitivity has a few maxima at 860 to 960 kHz, reflecting its design goal. Sharp sensitivity dips are found at 210 and 510 kHz. At 0.5 MHz, the nominal expected wavelength is 6 mm, which is probably close enough to the sensor element size causing the aperture nulling effect. The lower frequency sensitivity minimum cannot be accounted for by the same aperture effect.

#### A.3.3. PAC µ30D Sensor

This sensor, also from PAC, is a nominally 8 mm element diameter, 300 kHz design. Results of sensitivity tests are given in Figure A8. The averaged standard deviation among the three spectra increased 50% to 2.1 dB in comparison to S9220 sensor of the same size, shown in Figure A7. No plausive explanation can be found as the sensors were not opened for examining internal design details. Good sensitivity is found to 300 kHz, but it gradually diminishes as frequency increases. The sensitivity dip seen at 0.4 MHz corresponds to 8 mm wavelength for 3 mm/µs-wave velocity, leading to the aperture null effect.

#### A.3.4. PAC R15a Sensor

This sensor is an improved version of R15 sensor discussed above with 12.7 mm-diameter design for general AE applications. Figure A9 shows the results of sensitivity determination using plates and bars. The averaged standard deviation among the three spectra was 2.3 dB to 2 MHz. The sensitivity behavior was similar to that of R15 sensor. The sensitivity peaks appeared at 55 and 170 kHz, reaching 10 dB, but it dropped beyond 180 kHz as in R15 sensor, followed by sensitivity dips at 205 and 265 kHz, as well as at 665 and 1065 kHz.
